# Identification and development of microsatellite markers in *Hamamelis mollis* (Hamamelidaceae)

**DOI:** 10.1002/aps3.1189

**Published:** 2018-10-23

**Authors:** Qianyi Yin, Cuiying Huang, Yanshuang Huang, Sufang Chen, Huagu Ye, Qiang Fan, Wenbo Liao

**Affiliations:** ^1^ State Key Laboratory of Biocontrol and Guangdong Provincial Key Laboratory of Plant Resources Sun Yat‐sen University Guangzhou 510275 People's Republic of China

**Keywords:** conservation genetics, Hamamelidaceae, *Hamamelis mollis*, microsatellite marker, shotgun genome sequencing

## Abstract

**Premise of the Study:**

*Hamamelis mollis* (Hamamelidaceae) is a Tertiary relict species endemic to southern China. Polymorphic microsatellite markers were developed to reveal the genetic diversity of this species.

**Methods and Results:**

The genome of *H. mollis* was sequenced and de novo assembled into 642,351 contigs. A total of 72,097 paired primers were successfully designed from 80,282 simple sequence repeat (SSR) markers identified in 63,419 contigs. PCR amplification showed that 96 of the 136 synthesized primers could be successfully amplified, and 22 demonstrated polymorphism. The mean number of alleles, levels of observed heterozygosity, and levels of expected heterozygosity were 4.602 ± 0.140, 0.632 ± 0.020, and 0.696 ± 0.010, respectively. The majority of the 96 primer pairs could be amplified in at least one other Hamamelidaceae species, including *Distylium myricoides* (60), *Loropetalum chinense* (39), *Exbucklandia populnea* (24), and *E. tonkinensis* (24).

**Conclusions:**

These microsatellite loci provide abundant genomic SSR markers to evaluate genetic diversity of this woody ornamental plant.

Hamamelidaceae comprises six subfamilies and approximately 30 genera and 140 species distributed in subtropical to temperate regions of both the Old and New World (Li et al., 1999). The fossil record of Hamamelidaceae shows that the early members of this family were present during the Late Cretaceous of the Northern Hemisphere, yet many of them disappeared at higher latitudes due to global cooling during the Late Tertiary, which was further accelerated during Quaternary glaciation. Currently the family contains many isolated and diverse genera, most of which are Tertiary relicts (Zhang and Lu, 1995).

Subfam. Hamamelidoideae contains about 19 genera and 72 species. Within the subfamily, *Hamamelis* Gronov. ex L. consists of four to six species distributed disjunctly between eastern Asia (two species) and eastern North America (two to four species) (Wen and Shi, 1999). Fossil leaves of *Hamamelis* have been found from the Paleocene in both the Old and New World (Wolfe, 1966). The modern distribution of *Hamamelis* is much narrower at present than the wider past distribution indicated by the fossil record (Zhang and Lu, 1995). For example, *H. mollis* Oliv. is present today only in central and southern China (Zhang and Lu, 1995), where it occurs at elevations of 600–1600 m. It is often planted as an ornamental tree in China.

Knowledge of the genetic characteristics of species, such as genetic diversity and population structure, is vital for the management of effective conservation strategies for relict species. For subfam. Hamamelidoideae, some simple sequence repeat (SSR) markers have been developed for *Fothergilla* ×*intermedia* Ranney & Fantz (Hatmaker et al., 2015), *Chunia bucklandioides* H. T. Chang (Meng et al., 2016), *Distylium lepidotum* Nakai (Sugai and Setsuko, 2016), and *Sinowilsonia henryi* Hemsl. (Li et al., 2017), yet PCR amplifications have showed low transferability of these SSR markers to *H. mollis* (Q. Yin, S. Chen, Q. Fan, and W. Liao, unpublished). In this study, a total of 22 polymorphic genomic SSR markers were developed for *H. mollis* for further use in conservation measures. These are the first SSR primers designed for this species.

## METHODS AND RESULTS

Fresh leaves were collected from one seedling of *H. mollis* sampled from Juqiushui, Hunan Province, China (Appendix 1), and transplanted to the greenhouse of Sun Yat‐sen University, Guangzhou, Guangdong Province, China. The cetyltrimethylammonium bromide (CTAB) method of Doyle and Doyle (1987) was used to extract total genomic DNA. The DNA library was constructed with the VAHTS Universal DNA Library Prep Kit for Illumina (Vazyme Biotech Co., Ltd., Nanjing, Jiangsu Province, China) according to the manufacturer's protocol and was subsequently sequenced with the HiSeq X Ten System (Illumina, San Diego, California, USA). This yielded a total of 25.45 million high‐quality 145‐bp paired reads. Reads were filtered using NGSQCToolkit_v2.3.3 (Patel and Jain, 2012) by removing low‐quality reads (i.e., containing unknown bases [N] or more than 10% of nucleotides with Q value ≤20). Filtered reads were de novo assembled into 642,351 contigs using Edena v3.131028 with the default parameters (Hernandez et al., 2008). The average length of the contigs was 541 bp and the N50 value was 311 bp. The filtered raw data and the assembled contigs were deposited in the National Center for Biotechnology Information's (NCBI) GenBank database (BioSample: SAMN09010486, BioProject: PRJNA454742, Sequence Read Archive: SRR7110723, Transcriptome Shotgun Assembly: GGNJ00000000).

The SSR repeat motifs containing two to six nucleotides across these contigs were identified using the MISA tool (Thiel et al., 2003) with the default parameters except that mononucleotide repeats were removed from analysis. A total of 95,585 SSRs were found across 75,595 contigs. Among these SSRs, the most common motifs were dinucleotide repeats (76.75%), followed by tri‐ (17.70%), tetra‐ (3.89%), penta‐ (1.15%), and hexanucleotide (0.51%) repeats. Primer 3.0 (Rozen and Skaletsky, 1999) was used to design SSR primers with default parameters. This yielded a total of 72,097 primer pairs across 63,419 contigs. Of these primer pairs, 136 with high, medium, and low repeats were selected randomly for further characterization.

A total of 80 individuals of *H. mollis* were collected from four natural populations in China (Appendix 1) to test the levels of polymorphism in the target SSR loci. The transferability of these SSR primers was also tested in four other species of Hamamelidaceae (eight individuals of each species; Appendix 1): *Distylium myricoides* Hemsl. (subfam. Hamamelidoideae), *Loropetalum chinense* (R. Br.) Oliv. (subfam. Hamamelidoideae), *Exbucklandia populnea* (R. Br. ex Griff.) R. W. Br. (subfam. Exbucklandioideae), and *E. tonkinensis* (Lecomte) H. T. Chang (subfam. Exbucklandioideae). DNA was extracted from silica‐dried leaves of all individuals using the CTAB method described above. In the first PCR trial, all 136 primer pairs were amplified for two individuals randomly selected from each population. PCR amplifications were performed according to Fan et al. (2013) and were inspected using 10% agarose gel electrophoresis. Among all primer pairs, 96 were successfully amplified across the eight test individuals with the expected product size (NCBI accessions: MH167492–MH167587). The Fragment Analyzer Automated CE System (Advanced Analytical Technologies [AATI], Ames, Iowa, USA) was used for genotyping, using the Quant‐iT PicoGreen dsDNA Reagent Kit (35–500 bp; Invitrogen, Carlsbad, California, USA). Fragment sizes were analyzed using PROSize version 2.0 (AATI). The results showed 22 polymorphic loci across all eight individuals (Table 1) and 74 monomorphic loci (Appendix 2).

**Table 1:** Characteristics of 22 polymorphic EST‐SSR loci developed for *Hamamelis mollis*

Locus	Primer sequences (5′–3′)	Repeat motif	Product size (bp)	Allele size range (bp)	*T*_a_ (°C)	GenBank accession no.	Putative function
H4	F: ACACCTAATTCGCAGGCATC	(AAAAAT)_6_	215	191–227	60	MH167495	*Swertia tetraptera* microsatellite ST40 sequence
	R: ATGAAGTGGCATTCGGAAAC						
H7	F: TTGATGGGTTTTGTGGGAAT	(GAAAA)_8_	238	218–238	62	MH167497	PREDICTED: *Glycine max* homeobox‐leucine zipper protein HOX11‐like (LOC100805312), mRNA
	R: TGAACCACGGAACAAAACAA						
H11	F: TCAACCATGAGTGTGTACCTAGC	(ATAC)_11_	243	231–247	60	MH167500	*Chrysanthemum* ×*morifolium* microsatellite JH‐1484 sequence
	R: CCTCTAATCACAGGCAACCAA						
H22	F: CATGGGTTACGGCTGTCTTT	(ATC)_15_	237	217–250	60	MH167508	PREDICTED: *Ziziphus jujuba* uncharacterized LOC107420631 (LOC107420631), mRNA
	R: TGCTGGTACTAACCTTGGGG						
H52	F: ATGCCAAGGAGAGGGAAAAT	(AAT)_9_	184	172–181	60	MH167529	NOT FOUND
	R: GCTTTTTATGCTTTAGGTTTCTGC						
H54	F: AAACCGAAAGAAAGCACAACA	(AAT)_9_	222	208–219	60	MH167530	NOT FOUND
	R: GGGTTTTAAGCTTGCCATGT						
H57	F: CCTGGATAATGGAGAGCCAA	(TC)_23_	242	226–242	60	MH167533	PREDICTED: *Durio zibethinus* amino acid transporter AVT1I‐like (LOC111317757), mRNA
	R: TTTTTGTGTTGCATTACGTGC						
H63	F: TATATGCGCAGTGGAGCAAA	(GAGCAA)_6_	237	213–243	60	MH167537	NOT FOUND
	R: TGCCCATTAACACTGGTTCA						
H64	F: TCCAAGTAAAGGATCCGAACTC	(CTTTTT)_6_	251	233–257	62	MH167538	NOT FOUND
	R: TTGGCTATTGATGGTGCTTT						
H67	F: GATTTTGTGCATGTTTCCCC	(AGCCCA)_5_	236	212–242	60	MH167540	NOT FOUND
	R: AGGGGGTATCGGTGATTGTT						
H71	F: TGTCAACTGGAACATCAAGGA	(AAATA)_6_	255	240–260	60	MH167543	NOT FOUND
	R: TGTTTCTGAGTGTCCCAACCT						
H77	F: CCAGCTTGGAGTACACATGG	(AC)_18_	174	164–178	60	MH167548	NOT FOUND
	R: GAGGGATGCCTTTAACACCA						
H86	F: ATAGCAGAACCAGGCACCAG	(AAG)_12_	274	265–283	65	MH167555	NOT FOUND
	R: TTCATTAGTCACCGGAAGGC						
H94	F: TGGAAAACGGACAGAGTGAA	(AAAT)_5_	225	217–229	60	MH167560	NOT FOUND
	R: GCCATTCATTGGCTTTTTGT						
H99	F: ATCGCTAACCCGCTCCTAAT	(CCAGG)_6_	250	220–250	60	MH167561	NOT FOUND
	R: TTCAGCTAGCAAATAAGATTGACC						
H103	F: GAATGCATGTGACTGATGGG	(CTTTT)_6_	220	210–225	60	MH167563	NOT FOUND
	R: TTGCTTTCCTTTTCCATTGC						
H115	F: ATGGGCGAAAAAGATTGTTG	(CTC)_12_(CTT)_5_	237	222–240	62	MH167570	NOT FOUND
	R: GCCTTCACGTCCTCACAAAT						
H122	F: GTTTGGACACGCTCGTCATA	(AAT)_8_…(AAT)_6_	211	211–232	60	MH167575	NOT FOUND
	R: CCATCTCTGTCCTTGCATGA						
H126	F: TGAAAGAAACGTCACCCTCC	(TCT)_7_…(GAA)_5_… (AAG)_5_	279	264–282	60	MH167579	*Botryotinia fuckeliana* T4 SupSuperContig_200r_370_1 genomic supercontig
	R: GATCGTCATCATCACAACCG						
H130	F: GGCCTTCCAACGGTCATATT	(TTGAGT)_5_…(TTTGAG)_5_	258	263–282	62	MH167582	NOT FOUND
	R: AGGGAGGCATGTCAATTCAT						
H131	F: GGGAAAAAGAAGAAGGAGAAGG	(AGA)_10_	255	246–270	60	MH167583	NOT FOUND
	R: GCCTTGTTTGGCATTGAACT						
H132	F: GCATTTGGTTGCGGTTAGAG	(TAT)_10_	272	266–287	60	MH167584	NOT FOUND
	R: TCTACCAGGGGTGGAAGAGA						

Note

*T*_a_ = annealing temperature.

For these 22 polymorphic SSR loci, PCR amplification was performed for all individuals in the four populations of *H. mollis*. Linkage disequilibrium, departure from Hardy–Weinberg equilibrium (HWE), the average number of alleles per locus (*A*), the observed heterozygosity (*H*_o_), and the expected heterozygosity (*H*_e_) were calculated using GenAlEx version 6.5 (Peakall and Smouse, 2012). No pairs of loci showed linkage disequilibrium after a sequential Bonferroni correction for multiple tests, indicating that the 22 markers can be considered independent markers. Significant deviations from HWE were detected in five loci in the DLL population and four loci in the YS population (Table 2). In the DLL population, *A* ranged from three to eight, *H*_e_ ranged from 0.515 to 0.838, and *H*_o_ ranged from 0.000 to 1.000. In the TMS population, *A* ranged from three to eight, *H*_e_ ranged from 0.445 to 0.829, and *H*_o_ ranged from 0.000 to 0.858. In the WGS population, *A* ranged from three to seven, *H*_e_ ranged from 0.445 to 0.788, and *H*_o_ ranged from 0.000 to 0.850. In the YS population, *A* ranged from two to six, *H*_e_ ranged from 0.320 to 0.788, and *H*_o_ ranged from 0.000 to 0.950.

**Table 2:** Polymorphism in 22 SSR loci across four populations of *Hamamelis mollis*.^a^

Locus	DLL (*n* = 20)			TMS (*n* = 20)			WGS (*n* = 20)			YS (*n* = 20)		
	*A*	*H*_o_	*H*_e_ b	*A*	*H*_o_	*H*_e_ b	*A*	*H*_o_	*H*_e_ b	*A*	*H*_o_	*H*_e_ b
H4	4	0.65	0.744	5	0.50	0.641	7	0.55	0.715	5	0.60	0.734**
H7	3	0.45	0.540	5	0.70	0.759	5	0.65	0.731	5	0.70	0.773
H11	3	0.40	0.515	3	0.50	0.624	3	0.55	0.660	2	0.45	0.439
H22	8	0.55	0.853*	8	0.65	0.784	6	0.65	0.788	5	0.70	0.788*
H52	4	0.50	0.629	3	0.65	0.660	3	0.55	0.654	3	0.55	0.595
H54	5	0.45	0.665**	4	0.70	0.726	5	0.70	0.781	4	0.55	0.716
H57	7	0.60	0.775	5	0.86	0.761	6	0.70	0.775	3	0.65	0.635
H63	5	0.60	0.665	3	0.70	0.636	5	0.75	0.736	4	0.55	0.648*
H64	5	0.85	0.775	4	0.70	0.741	4	0.85	0.696	4	0.60	0.701
H67	6	0.65	0.771	6	0.65	0.749*	5	0.65	0.739	5	0.75	0.709
H71	3	0.55	0.614	4	0.55	0.671	4	0.70	0.729*	3	0.50	0.609
H77	7	0.60	0.838	7	0.65	0.759	5	0.60	0.726	5	0.55	0.741
H86	5	0.65	0.706	4	0.70	0.685	4	0.60	0.648	4	0.65	0.736
H94	4	0.55	0.644	3	0.60	0.651	4	0.55	0.643	3	0.65	0.629
H99	5	1.00	0.693*	5	0.90	0.709	4	0.80	0.705	5	0.65	0.673
H103	3	0.55	0.609	4	0.75	0.738	4	0.60	0.729	4	0.70	0.748
H115	7	0.55	0.719	6	0.75	0.765	6	0.65	0.813	5	0.80	0.738
H122	6	0.95	0.779*	6	0.85	0.829	7	0.85	0.808	6	0.95	0.765
H126	4	0.60	0.559	7	0.80	0.829	5	0.70	0.769	5	0.75	0.765
H130	3	0.00	0.645***	3	0.00	0.445***	3	0.00	0.445***	2	0.00	0.320***
H131	5	0.55	0.756*	4	0.70	0.679	5	0.75	0.726	4	0.65	0.701
H132	4	0.85	0.686	5	0.75	0.711	5	0.75	0.735	4	0.80	0.636

Note

*A* = number of alleles; *H*_e_ = expected heterozygosity; *H*_o_ = observed heterozygosity; *n* = number of individuals collected for each population.

^a^Voucher and locality information are available in Appendix 1.

^b^Significant deviations from Hardy–Weinberg equilibrium after sequential Bonferroni corrections: *represents significance at the 5% nominal level; **represents significance at the 1% nominal level; ***represents significance at the 0.5% nominal level.

Finally, transferability tests indicated that the majority of the 96 loci could be amplified in at least one other Hamamelidaceae genus. Specifically, we found that 60, 39, 24, and 24 paired primers amplified in *D. myricoides*,* L. chinensis*,* E. populnea*, and *E. tonkinensis*, respectively, and 24 amplified in three of the four species (Table 3).

**Table 3:** Cross‐amplification success and fragment size ranges (in base pairs) for 96 SSR markers in four Hamamelidaceae species.^a^

Locus	*Distylium myricoides*	*Loropetalum chinense*	*Exbucklandia populnea*	*Exbucklandia tonkinensis*
H1	159	177–183	171–177	189
H2	—	—	—	—
H3	234–240	—	—	—
H4	197	233	—	—
H5	155	101	125–137	131–143
H7	—	—	—	—
H8	—	—	—	—
H10	227	—	—	—
H11	—	203	—	—
H13	—	—	—	—
H14	232–240	224–228	—	—
H15	232–236	—	—	—
H17	208	172–176	192	192–196
H18	207	—	—	—
H19	218–224	248	197	197
H20	—	255	—	—
H22	268–271	—	196	190
H23	243	225–228	168	168
H25	292–295	303	253–256	262
H26	195–198	234	246	246
H27	254–263	278–281	293	272–275
H28	199	—	—	—
H33	254–258	266	286–290	294
H34	114	—	—	—
H36	—	—	—	—
H38	308–312	256–264	—	—
H39	276–280	237	241–245	245
H40	222	262	—	—
H41	246–250	202–206	214–218	210
H42	248	—	—	—
H43	—	—	—	—
H44	201	—	—	—
H46	—	—	277	265
H47	291–297	264–270	—	—
H48	—	287	—	—
H49	200–203	—	—	—
H50	—	—	—	—
H52	—	145	—	193
H54	199	234	—	—
H55	—	—	—	—
H56	188	—	—	—
H57	210	—	—	—
H58	208	232	174	174
H59	223	—	—	—
H62	245	203	—	—
H63	207–212	—	—	—
H64	—	—	—	—
H65	—	—	106–109	122
H67	—	—	—	—
H68	300–305	245	—	—
H70	297–302	282	242	242
H71	220	—	—	—
H72	242–252	232	—	—
H73	183	—	—	—
H74	—	—	—	—
H76	277–281	233	—	—
H77	—	—	—	—
H79	—	—	—	—
H80	212–216	164–166	154	154
H81	269	281–283	213	221–223
H83	—	125–129	—	—
H84	—	218	256	252–256
H85	—	—	—	—
H86	—	—	—	—
H88	154	—	—	—
H91	267–271	—	—	—
H92	233–241	—	225	225
H93	243–247	—	—	—
H94	197	—	—	—
H99	—	—	—	—
H100	131	—	—	—
H103	—	—	—	—
H105	214–219	244	—	—
H108	—	200	—	—
H110	—	—	—	—
H111	195–199	231–239	227	227–235
H112	242–250	212	—	—
H113	—	—	—	—
H115	204	—	—	—
H117	136–140	176	—	—
H118	299	—	—	—
H119	254–258	266–270	—	—
H121	233	—	—	—
H122	—	196–199	—	—
H123	—	—	—	—
H124	—	—	—	—
H125	235–238	—	—	—
H126	—	—	—	—
H127	275–281	—	—	—
H129	—	219	—	—
H130	221–233	—	245	251
H131	—	—	—	—
H132	—	—	—	—
H133	—	—	—	—
H135	241–250	—	292–195	301–307
H136	108	—	165	—

Note

— = primers could not be amplified in any individual.

a Voucher and locality information are available in Appendix 1.

## CONCLUSIONS

We have developed a number of useful new primers for assessing genetic diversity in *H. mollis* and potentially numerous other Hamamelidaceae taxa. These loci will aid in future conservation genetics efforts across the family.

## AUTHOR CONTRIBUTIONS

W.B.L. and Q.F. designed the research. Y.S.H. and H.G.Y. collected samples. C.Y.H. designed the primers. C.Y.H. and Q.Y.Y. generated the data. Q.Y.Y. and S.F.C. analyzed and interpreted the data. Q.Y.Y. wrote the manuscript, and S.F.C. modified the manuscript.

## DATA ACCESSIBILITY

All genomic sequences of 136 pairs of primers were deposited in the National Center for Biotechnology Information (NCBI) GenBank database (MH167492–MH167587). The filtered raw read data and the assembled contigs were also deposited in NCBI databases (BioSample: SAMN09010486, BioProject: PRJNA454742, Sequence Read Archive [SRA]: SRR7110723, Transcriptome Shotgun Assembly [TSA]: GGNJ00000000).

## Appendix 1 Voucher and locality information for populations of *Hamamelis mollis* and four related Hamamelidaceae species used in this study.

**Table:**

Species	Voucher no.^a^	Collection locality (Population)	Geographic coordinates	*N*
*Hamamelis mollis* Oliv.	W. Y. Zhao et al. 16871^b^	Yanling, Hunan, China	26°33'13.4”1” N, 114°04'51.55” E	1
	Q. Fan et al. 15216^c^	Yichang, Hubei, China (DLL)	31°04'15.37” N, 110°55'40.56” E	20
	Q. Y. Yin et al. 17161^c^	Lin'an, Zhejiang, China (TMS)	30°48'34.67” N, 120°55'55.06” E	20
	Q. Y. Yin et al. 17277^c^	Pingxiang, Jiangxi, China (WGS)	27°21’46.82”N, 113°46’3.50” E	20
	Q. Y. Yin et al. 17193^c^	Shaoguan, Guangdong, China (YS)	25°22’5.66”N, 114°35’32.30” E	20
*Distylium myricoides* Hemsl.	X. J. Zhang et al. 17009	Yichun, Jiangxi, China	28°34’29.62”N, 114°35’32.30” E	8
*Exbucklandia populnea* (R. Br. ex Griff.) R. W. Br.	W. Y. Zhao et al. 16235	Tongren, Guizhou, China	27°57'55.32” N, 108°36'46.67” E	8
*Exbucklandia tonkinensis* (Lecomte) H. T. Chang	W. Y. Zhao et al. 16339	Zhaoqing, Guangdong, China	23°33'31.93” N, 111°57'52.00” E	8
*Loropetalum chinense* (R. Br.) Oliv.	Q. Fan et al. 17439	Huangjiang, Guangxi, China	25°12’9.82”N, 108°38’23.94” E	8

## Appendix 2 Characteristics of the 74 monomorphic SSR markers in *Hamamelis mollis*.

**Table:**

Locus	Primer sequences (5′–3′)	Repeat motif	Expected allele size (bp)	GenBank accession no.
H1	F: GTTGCTTTCGTGTTCGTCCT	(CTCGTC)_9_	171	MH167492
	R: GTTTGGTAAGGCAAGGGACA			
H2	F: CCTCCATATCGTAGTCTACCGC	(TTTTGA)_8_	255	MH167493
	R: TTCTACCACACGTCACACCC			
H3	F: CTCGACGACTTTTGGTGGAT	(TTTCTC)_7_	246	MH167494
	R: CCCAATGAGGCTTTGAAAAA			
H5	F: AGCATTGAATGTTGCGTTTG	(CTCTTC)_5_	119	MH167496
	R: CTACGGGGGACAGCAGAATA			
H8	F: TTTTGCCCTTTCTCTCCCTT	(TTCTC)_7_	261	MH167498
	R: TGTTTGGATTGAAGGAATTGG			
H10	F: TGAAAGAGAAGGGAATGGCA	(CTGCC)_5_	252	MH167499
	R: TTTTTGTCCAATTCATGGCA			
H13	F: TGTGGGGCGAGGATAAATAG	(AAAT)_9_	232	MH167501
	R: GGGGAGAGGACGAGGAATAG			
H14	F: CCGTTGCAATCCCTGTAGTT	(TTTC)_8_	248	MH167502
	R: CAATTTTGGCTGCAATTCAA			
H15	F: GTGGTAAAATTGGGTGCTGG	(TTTA)_7_	216	MH167503
	R: TCGTGGTCGTCTAAGTCACG			
H17	F: ACTCTTGTTCCCCCACCTTT	(ATTA)_5_	184	MH167504
	R: GTAACCCAGGATGACCCCTT			
H18	F: TATCCCTCGCTTGATTTTGC	(ATA)_19_	195	MH167505
	R: GCAATAGAGCTCGACGGTTC			
H19	F: AGCAAGATGGAAGGAAGCAA	(TTC)_18_	230	MH167506
	R: AAACCTATCATGCATACTAACAATGAA			
H20	F: GAAAGGCAACAGAGCTCGAC	(TAT)_17_	279	MH167507
	R: CCAACAGTCGGATCAATGTG			
H23	F: TATCCACCCCACTCCAATTC	(AAT)_14_	201	MH167509
	R: CCATTTCTTGCAGGTTTGCT			
H25	F: GCCTGGTTATTTTTGGAAACTT	(ATA)_9_	277	MH167510
	R: TGTGTGCGCACTTAGGTGAT			
H26	F: GTGTCCGCACTTCATAGGGT	(TTG)_8_	219	MH167511
	R: CGCCTCCTTAACTGCATACC			
H27	F: GCTCACTAACTCTGCCTGGG	(CAT)_5_	266	MH167512
	R: TTCCGGAAAGCCAGTCATAC			
H28	F: ATTCTGCTTTGGACCTGCAT	(ATT)_5_	172	MH167513
	R: TGCTCATACAAATGTCCCAAA			
H33	F: CCTTGGTTTCCCTCATTTCA	(TC)_19_	272	MH167514
	R: TGAATCTTGTGGTTCGTCCA			
H34	F: TTTACTTGGGGACTTGGGAA	(TA)_10_	100	MH167515
	R: ACAAGAGTCCTGAAGTTTGAATGA			
H36	F: CATAGTAAAAACACATTGAACACACTG	(TA)_7_	257	MH167516
	R: TTGACAGTAAAAATACTAAAAATGGTG			
H38	F: CAACGGAATTCAAAAATCTCG	(TTAT)_9_	276	MH167517
	R: CGCTGCAATGTTCATACGAC			
H39	F: CAACCCCTCTCCCCTCTAAA	(TACA)_9_	253	MH167518
	R: GGGTCCGTTGGTTTTAGCTT			
H40	F: ACGGGTTTAAGCGCTAAGGT	(TATG)_8_	246	MH167519
	R: TTGAAGGGGAAAATGTGCTC			
H41	F: AAGCCACATGCCAAGTTTTC	(AAAC)_7_	222	MH167520
	R: TTGTTTTGAAGGTTGGGTCA			
H42	F: AAGGCATTGCTGTCATTTCC	(TATG)_7_	274	MH167521
	R: TCCTTCAAAGACCCCGTACA			
H43	F: TCGAAGAAAAAGCTGGAAGC	(TTC)_15_	177	MH167522
	R: ACTTAGGTACCCATCCCTATCAT			
H44	F: AAAAACAACACCCAACCCAT	(TTA)_15_	183	MH167523
	R: GGAGTTGGAATGCCTTTGTC			
H46	F: TCAAAATTGATGTGGCACTAGC	(ATT)_14_	232	MH167524
	R: CAAGGGAATTTTGTTGGCAT			
H47	F: TGGCATCATTTTACTTTCTAAGCA	(TAA)_14_	279	MH167525
	R: TGATGGGACTCAATCACTTTG			
H48	F: CAAAGCCTCAATGATGACGA	(TAA)_14_	264	MH167526
	R: TGAAGGGTTCAAAAAGAGATGAA			
H49	F: TCCTTTGCATACTAGGGAAATAAAA	(AAG)_13_	188	MH167527
	R: CTTGGAGTCCTTGGAGCTTG			
H50	F: GAGGGAGCATAGCAAAGGTG	(TTA)_13_	221	MH167528
	R: TCAAATGTGGACCTTAAATCACTC			
H55	F: TCTCCCGATTTGAGGGTATG	(GA)_25_	217	MH167531
	R: ACGTCATTGCGAGTCCTCTT			
H56	F: CGAGAAAGGTCAAAGGTGGA	(GA)_25_	164	MH167532
	R: ATTGCAAAACGAAGCCTCTG			
H58	F: TCTTAAAGGGTCAATGGGCA	(TC)_23_	220	MH167534
	R: AATCACACAACACCGCCTTT			
H59	F: CGCTAATGCGCATCTGTACT	(TC)_23_	245	MH167535
	R: TTGGAAAACCTGCTCGATCT			
H62	F: TGCCTTTGCTTGTTATGTTGTC	(TTGCCT)_7_	227	MH167536
	R: TCGATACCAAATGAGGGCAT			
H65	F: TTGAAAGCAGGAAATGGACA	(ATAAAA)_5_	127	MH167539
	R: AAAGTAGTGACCCCCGTCCT			
H68	F: AACCAATTGAAAAGAAAAGAACG	(CTTTT)_7_	280	MH167541
	R: GACTCCCTAATGTCGGCAAA			
H70	F: GGTCAACAGAAATATGGCCC	(TGAGT)_6_	272	MH167542
	R: GATGCCTGTGGTCTCTGGAT			
H72	F: TATCACGACTTTGTGCCTGC	(TTTTC)_5_	222	MH167544
	R: TGCTAGCAATGCTTTCCGAT			
H73	F: GCCCGATAATCTCAACTGGA	(TTTCT)_5_	203	MH167545
	R: TGGCTGCCTAGCTAACACCT			
H74	F: CATCCTTATCCACCCACCAC	(TATTA)_5_	114	MH167546
	R: AACGAAGGAGCGTAGTGTCG			
H76	F: CATTGCCAAATTTGAGAGCA	(AAAC)_6_	245	MH167547
	R: TCTAAACAATTCGTTCGGGC			
H79	F: TCCGATTAAAAACTGCCACC	(CT)_15_	268	MH167549
	R: ATATTTCCCAGCGTGTCAGG			
H80	F: CTTTGCCTGAATGGCTGAAT	(CT)_15_	182	MH167550
	R: TTCAATAGGCAAGCAATCCC			
H81	F: GTCGAGAACACTTCCATGCC	(TG)_24_	251	MH167551
	R: TACGTCACCCCGTAAGATCC			
H83	F: TTGTGGTTCTTATGGCCCTC	(AG)_21_	147	MH167552
	R: AGCTTCACTTGCCTCATCGT			
H84	F: CACACCTGCAAAATCACCAC	(AG)_21_	240	MH167553
	R: ATTTGATACATTGGCGAGGC			
H85	F: CCTGCCGTTGCAATAACTCT	(AT)_11_	169	MH167554
	R: TGGTAGACATGGCATCCGTA			
H88	F: ATGACCTTCAACAGGCACAT	(TAT)_16_	181	MH167556
	R: CATTGCAATCAAAATCGTTCA			
H91	F: TAGTTGATTGGCTCCCTTGG	(TCTA)_7_	239	MH167557
	R: CCCTCTCGCTATGTTTCTGC			
H92	F: CGTGGGATCAAGGGAAGTAG	(CAAC)_7_	217	MH167558
	R: GGATGTGACTGTGCTTCCAA			
H93	F: GGGCAATGTCCCTTCTTGTA	(AAAT)_5_	231	MH167559
	R: AGAATTGTTAGGGCCGGTTT			
H100	F: CTGCAAATCTTGGTTTGGCT	(TTTCC)_5_	146	MH167562
	R: AATTCCCCAGAAAAGGTTCG			
H105	F: TTAGGCTCCGTTTGGTTGTC	(AGGAA)_5_	234	MH167564
	R: GTGGGAAAATTGTGGACCAA			
H108	F: TTTGGTTGAGTGGGACTTGA	(TTGG)_8_	228	MH167565
	R: CAAGGGACTGGAGCTCAAAC			
H110	F: TCAAGACCTTTTACTCCCAAAAA	(AAAG)_8_	122	MH167566
	R: AAAGAGCATCGTGGCTAAAGTC			
H111	F: GCATTGGAGGAATACGGTTG	(ACAT)_7_	219	MH167567
	R: GGAGCAGAAATTCCACGAAC			
H112	F: TGTCCTTTTGGTGTTTTCACA	(TTTA)_7_	230	MH167568
	R: AATTCACTGTCACCATCCCC			
H113	F: TTCAGTTTCTTTGTTGGGGC	(ACAT)_7_	219	MH167569
	R: ATCCAACGCCTCCTAATCCT			
H117	F: GAGTGCGTACACGGGTTCTT	(TTC)_6_…(TTC)_5_	152	MH167571
	R: CCCATCTAGTTCCTTCTCTTTGC			
H118	F: GGGTGAAGATTTTGGATTCG	(TA)_9_(CA)_8_	263	MH167572
	R: AAATCACAGCCACAGAGTGAGA			
H119	F: GCCCTATAGAGGTCACCTTCC	(AC)_12_(AT)_12_	272	MH167573
	R: CTCACCCGAAAGCCATAAAA			
H121	F: TTTTGAGAACCAAAATAGAGTATAGCA	(AAC)_9_(AAT)_8_	257	MH167574
	R: TTGGAACTCATCAGTTTTTCCA			
H123	F: ATCACTGCTAGTCCGCCACT	(CTG)_6_GTTCTGG(TTC)_8_	155	MH167576
	R: AGGAACCGGGAAAAGAAGAA			
H124	F: GACGAGCGTACCCTTCAAAA	(GAA)_8_…(AGA)_7_	156	MH167577
	R: GCGACGCAGTGGTCTTCT			
H125	F: GCCAAGTAGCCGACTTTGAA	(TTA)_10_TTTAT(TTA)_6_	268	MH167578
	R: CTGCTCAACTCAACAAAGCCT			
H127	F: AGCCTCAAGGCATTACACCA	(TCT)_5_TATTCTTA(TTC)_7_TTT(TTC)_6_	263	MH167580
	R: GGTACCTATCCCTAGCATGGC			
H129	F: ATGCAGAAATGCCCTTGCTA	(ATT)_9_…(ATT)_9_	228	MH167581
	R: GCAATTACAGGTAAAGCTAATCCAA			
H133	F: AGAGGTGGTGTTCAAAACGG	(TTA)_10_	200	MH167585
	R: TGGCATACCTAAAATCCTAAATCA			
H135	F: GGCTAAAGTGAGGTTTTGGC	(ATT)_14_	277	MH167586
	R: GCTCCGAGCTAACAAAGCAC			
H136	F: TGCAACTAATCCTAACCTTTGAA	(GAA)_14_	132	MH167587
	R: AGGAGCAAAGGAGAAGGGAG			
